# Service, Prof. E. Andrews, M. D., Attending Surgeon

**Published:** 1861-11

**Authors:** 


### CHICAGO MEDICAL AND SURGICAL DISPENSARY.

Service, Prof. E. ANDREWS, Attending Surgeon.

Reported for the EXAMINER.

*Gun-Shot Wounds.*—GENTLEMEN :—This patient is a soldier in Col. Mulligan's regiment, and was shot at the siege of Lexington. You observe that the bullet has traversed the forearm obliquely, cutting both the radius and ulna, one of the orifices being near the middle of one edge of the forearm, and the other on the opposite edge of the wrist. I will question him, and endeavor to ascertain from his position, at the moment of injury, which was the orifice of entrance, and which of exit.

*Question.*—Where were you when you were wounded?

*Answer.*—At Lexington, Sir.

*Q.*—What were you doing when you were shot?

*A.*—We were coming back from the hospital, Sir.

*Q.*—How is that?

*A.*—Why, you see, the hospital was outside of the lines, and they brought us word that the enemy was killing the surgeons and the chaplain, so we went out and drove them off, and bayonetted a dozen of them, and coming back, I was shot as I held my arm so, (*showing the position*).

Gentlemen, this man's allusion to the fight at the hospital, may justify me in a moment's digression, to render a tribute to the bravery of the surgeons in that unfortunate fight. Two of them from this city, Surgeons PARKER and WINER, I personally know to be worthy of all praise. The hospital was, as this man says, outside of the lines. The enemy at first fired on it, with red-hot shot, with the intention of burning it. Men were cut to pieces by the balls, and even the wounded were shot as they lay on the floor. Subsequently, the enemy took possession, and made the surgeons prisoners. At this juncture, Col. Mulligan's men made a charge with the bayonet, and cleared the building of the enemy, piling the halls with dead, and making the floor swim with blood, but the enemy rallying in greater numbers, retook the place,—driving out our men, and on the retreat, this soldier was wounded. During all this time, our surgeons were exposed to the fire, and continued, without flinching, to stand to their duty, calmly dressing the wounded, among the bullets and red-hot shot.

From the position of the parties, you see that the ball must have entered at the upper orifice and came out at the radial side of the wrist. It therefore fractured both the radius and ulna, and severed a number of important organs. By questioning, I perceive that he has no sensation in the little finger, nor in the ulnar side of the ring finger. This shows that the ulnar nerve was cut; that the flexor tendons of most of the fingers were severed, is manifest, by the loss of motion in all except the index finger and the thumb.

Gun-shot wounds are remarkable for the amount of shock they produce. This man says he had a severe chill for three hours after the wound was received. In bullet wounds of the extremities, the shock does not of itself prove fatal, but when a limb is torn and shattered by a cannon ball, it is not uncommon for the patient to die without reaction. As the wounds are lacerated and contused, there is seldom any considerable hæmorrhage. Even the thigh has been cut off by a cannon ball without serious loss of blood. Prof. PALMER, surgeon of the 2d Michigan regiment, informs me, that at the battle of Bull Run, he sent his assistant, under fire, armed with tourniquets, ligatures, etc., for the exprcss purpose of promptly suppressing hæmorrhage, but, that out of fifty wounded, not a single case required that kind of assistance, as there was no serious loss of blood in any instance. In fact, he deemed the common instruction, to send a portion of the assistant surgeons with the line of battle, to be a useless risk of life to these medical officers. His advice is, that the wounded should all be brought into the field depot for attendance. I cannot wholly agree with these conclusions, because it is well proved that gun-shot wounds do, in some instances, bleed alarmingly, and require instantaneous attention. I had one case where the ball cut one side of the subclavian vein, and well nigh drained out the life of the wounded man, before I reached him. In the French army, in the Crimea, this matter was well understood, and a certain number of tourniquets were distributed among the soldiers, with instructions how to use them in emergency. This benevolent effort was brought into ridicule and disuse, by the blunder of one of the soldiers. The man observing a comrade wounded, and bleeding dangerously from the jugular vein, *applied his tourniquet around the neck.* Fortunately, he observed the alarming effects upon the respiration, and loosened it in time to avoid fatal asphyxia.

Gun-shot wounds are often clogged with foreign bodies, such as buttons, pieces of clothing, gravel, sticks, fragments of bone, and the projectile itself. These, of course, are to be removed whenever possible. Besides the objects just enumerated, there is a stratum of dead and contused flesh, surrounding the track of the ball, which is virtually a foreign body. From this fact, you will readily perceive, that a union of the wound, by first intention, is out of the question. The stratum of dead tissue, decomposes and gives rise to foul, offensive, and often irritating discharges, and the amount of suppuration is very great. In fact, the unhealthy appearance of gun-shot wounds, induced the belief, formerly, that there was something poisonous in the gunpowder, which envenomed the bullet. This idea is now exploded, and the mechanical peculiarities of the injury, fully explain its destructive character.

The best treatment for this class of wounds, is usually a coldwater dressing. Straps and sutures are useless, and poultices are injurious. The latter confine the pus, and decaying shreds of flesh in the wound, and by favoring their decomposition there, cause erysipelas. Besides, they are troublesome to prepare, and require too much attention, which is no small consideration in military surgery, where an excess of labor comes on the hospital attendants. Water dressings, on the contrary, are easily applied, they are always attainable, they keep the wound clean, and finally they are powerfully antiphlogistic. For these reasons, military surgeons hold them in high esteem, and the results justify their encomiums. As this wound implicates the bones, it will be necessary to apply splints, as in any other fracture. Undoubtedly, the rotary motion of the forearm, will be lost, and the hand very much stiffened and impaired in its use, but it cannot be avoided. Such are the chances of war, and we can only commend this brave fellow to the rewards of a good conscience, and to the relief of a United States' pension.

